# The effects of ß-alanine on body composition and performance measures in collegiate females

**DOI:** 10.1186/1550-2783-9-S1-P2

**Published:** 2012-11-19

**Authors:** Amanda Buckley, Abbie Smith, Chelsey Scoggins, Craig Jones, Josh Holt, Elizabeth Sillasen, Brooke Cox, Stacie Urbina, Bill Campbell, Cliffa Foster, Lem W Taylor, Colin D Wilborn

**Affiliations:** 1 University of Mary Hardin-Baylor, Human Performance Lab, Belton, TX 76513, USA; 2 University of North Carolina Chapel Hill, Chapel Hill, NC 27599, USA; 3 University of South Florida, Exercise & Performance Nutrition Lab, Tampa, FL 33544, USA

## Background

ß-alanine has ergogenic potential based on its relationship with carnosine. Carnosine is rapidly degraded into ß-alanine and histidine as soon as it enters the blood. So there is no advantage to using direct carnosine supplementation. Previous studies have demonstrated that taking ß-alanine orally is effective at increasing intramuscular carnosine levels. The resistance training athlete may experience a higher training volume. This proposed benefit would increase work capacity and decrease time to fatigue. Therefore, the purpose of this study is to evaluate recreationally active collegiate females, following an 8 week strength training program while consuming either ß-alanine (BA) or placebo (PL) for body composition and performance changes.

## Methods

Sixteen collegiate females (21.0±2.19 yrs, 64.76±8.50 kg, 164.98±6.97 cm, 30.11±5.08 %BF) participated in a double blind placebo controlled strength training and supplementation study. Supplementation consisted of either 5 g maltodextrin or 3.4g BA (Dymatize Nutrition, Farmers Branch, TX), taken 15minutes prior to training. In addition, all subjects were given a post workout protein supplement of ISO-100 (Dymatize Nutrition, Farmers Branch, TX). All subjects were tested at baseline (T1), 4 weeks (T2), and 8 weeks (T3) over the 8 week supplementation study. Training consisted of 4x weekly upper and lower body resistance training. Body composition variables lean muscle mass (LBM), fat mass (FM), and percent body fat (BF) were assessed using DEXA. Performance variables VO_2max_ (VO2), aerobic time to exhaustion (TTE), wingate peak power (PP), wingate mean power (MP), bench press 1RM (BPmax) and repetitions at 65% (BPreps), leg press 1RM (LPmax) and repetitions (LPreps), vertical jump (VJ), and standing broad jump (BJ) were assessed using standard NSCA guidelines. Statistical analyses utilized separate two-way repeated measures ANOVA [time (T1 vs T2 vs T3) × group (PL vs BA)] for all dependent variables. 95% confidence intervals were also run for each variable.

## Results

There were no time × group interactions (p>0.05). Body composition (LBM, FM, BF) improved over time (p<0.01) for both groups. Maximal strength demonstrated a significant increase (p=0.001), and VJ increased at each time point (p=0.047). Confidence interval data demonstrated a significant increase in VJ and BJ for the BA group only from T2 to T3.

## Conclusions

Results from this study suggest that 4x weekly moderate intensity training is effective for increasing body composition and strength. BA supplementation may provide some additional benefit under periods of long duration (4+weeks) training on anaerobic power in women. These findings show 4 d/ wk of moderate intensity training, in conjunction with BA supplementation, demonstrated no advantage on strength and body composition. However, as a potential result of increased training volume and power, a longer BA and training regiment may have a small advantage on sports performance including vertical and broad jumps, in college-aged women.

